# Is Magnesium Supplementation an Effective Nutritional Method to Reduce Stress in Domestic Pigs? A Systematic Review

**DOI:** 10.3389/fvets.2020.596205

**Published:** 2021-01-12

**Authors:** Emily V. Bushby, Louise Dye, Lisa M. Collins

**Affiliations:** ^1^Faculty of Biological Sciences, School of Biology, University of Leeds, Leeds, United Kingdom; ^2^School of Psychology, University of Leeds, Leeds, United Kingdom

**Keywords:** magnesium, pig, nutrition, swine, stress, aggression

## Abstract

In commercial pig production, stressful events are common and can have detrimental impacts on the pig's health and welfare, as well as on the performance of the farm. Supplementary magnesium may reduce stress, and subsequent harmful and aggressive behaviors, that occur during stressful events, such as regrouping. However, reports on the efficacy of this treatment are mixed. We aimed to systematically review the studies in which magnesium was given to pigs to examine the effects on measures of stress. Of the 16 studies included in the final corpus, 10 reported at least one statistically significant beneficial effect of supplementary magnesium on reducing stress. However, two studies found that magnesium significantly increased stress suggesting supplementary dietary magnesium may be harmful in some cases. Overall, there are a limited number of studies investigating the possible effect of magnesium on reducing stress in pigs, and although results were varied, the majority found beneficial effects of supplementary magnesium.

## Introduction

It is not uncommon for commercially farmed domestic pigs (*Sus scrofa domesticus*) to experience negative stress during their lifecycle. Acute stress (such as transportation or regrouping) and chronic stress (such as excessive heat or over-stocking for an extended period of time) can both be detrimental to the animal's health and welfare, and have economic impacts due to increased susceptibility to disease, increased mortality, poor meat quality, and poor performance (1–3). To understand how an environment, situation, or event is affecting an animal, stress can be assessed by measuring physiological, physical, and behavioral changes. Physiological measures of stress, such as heart rate or cortisol, have typically been the most common method of measuring a stress response in animals. For example, hair cortisol has been shown to be a potential marker for chronic stress (4), whereas blood and salivary cortisol changes much faster in response to acute stressors (5). However, whilst these measures assess the level of arousal of the individual, they do not indicate valence—the physiological changes observed can be the result of positive (excitement) or negative stress, making interpretation difficult. These physiological measures are more easily interpreted and more useful when used in conjunction with behavioral measures, allowing for the valence of the animal to be assessed (6–8). Physical changes like skin lesion scores can also be used. For example, in pigs, lesions on the main body are likely the result of fighting and aggressive interactions (9), whereas tail lesions often signs of non-aggressive harmful behaviors (10).

Harmful social behaviors, such as tail and ear biting resulting in ear and tail lesions, are often multifactorial with factors such as genetics, access to enrichment, and stocking density influencing the frequency and severity; however, they can also be exacerbated by stress (11). Acutely stressful events, such as transport or regrouping, can also lead to an increase in aggressive behaviors such as fighting, due to the disruption and subsequent re-establishment of the dominance hierarchy (12). Not only are these types of harmful and aggressive behaviors detrimental to the pigs' welfare but they can have a huge economic impact for the farmer or producer. Performance measures, including growth rate and reproduction (13, 14), are all negatively impacted by a high level of stress, as well as resulting damage and skin lesions increasing the risk of disease and mortality. Later, aggression before slaughter can cause carcass damage resulting in a penalty for the producer (15, 16), and higher stress levels have also been shown to negatively affect meat quality causing, for example, pale, soft, and exudative (PSE) meat that is unattractive to the consumer (17, 18).

Often, acutely stressful events are unavoidable in current commercial farming systems, such as key events that involve a change of environment or social structure, including weaning, regrouping (also known as mixing), or transportation, Therefore, research which focuses on improving the welfare of commercially farmed pigs, especially during these periods, is crucial for the animals and producer.

The five freedoms (19, 20) describe the basic needs of an animal to guard against poor welfare. The five freedoms are the freedom from hunger and thirst; freedom from discomfort; freedom from pain, injury, or disease; the freedom to express normal behavior, and freedom from fear and distress. These basic requirements should be met before other areas can be addressed to ensure a good, or even positive (21) welfare state is met. Providing a nutritionally balanced diet with access to water meets the most basic requirement. However, nutrition can also improve welfare beyond simply meeting the animals' basic needs. For example, providing a varied diet in terms of texture and taste, allowing a choice of diet, or providing the diet in an enriching and stimulating way will allow for the animal to express more of its natural behavior (22–24). Adding additional nutrients above the required level to maintain bodily function and growth, such as increased tryptophan (25) or fiber content (26), has also been shown to improve behavior, welfare, and performance. In farmed animal species, supplementary magnesium has been seen to improve productivity, including increased eggshell strength in aged laying hens (27), reduced weight loss in heat-stressed hens (28), improved growth rate in sheep (29), and reduced time between weaning and next oestrous cycle in pigs and dairy cattle (30).

As a vital mineral for mammalian function, magnesium acts as a co-factor for over 300 different enzymes and plays key roles in processes including ATP production and immune function (31–33). A large body of research also suggests that magnesium may play a role in reducing stress, anxiety, and depression in humans via multiple mechanisms including the serotoninergic, glutamatergic, and adrenergic systems (34). Multiple reviews have concluded that there is evidence for beneficial effects of magnesium despite the poor quality of some experimental research [for reviews see: Stress and anxiety: Phelan et al. (35) and Boyle et al. (36); Depression: Derom et al. (37) and Eby and Eby (38)]. In commercial pig production, magnesium may be added to pig feed during a stressful event in an attempt to alleviate this (39, 40). Swine diets typically contain sufficient magnesium to maintain growth and normal bodily function due to the level of magnesium in the cereal components of the feed; however, supplementation can be implemented with a range of different magnesium compounds or products. Although magnesium is generally thought to be beneficial in reducing stress, there remains a lack of substantive evidence to support its effectiveness in pigs.

Our aim was to conduct a systematic review to evaluate the available scientific evidence and determine whether this supports the use of supplementary dietary magnesium as an intervention to reduce stress in pigs. Included papers could focus on chronic or acute stress but must include a dietary magnesium treatment and at least one measure of stress, for example physiological measures such as cortisol, adrenaline, and heart rate, skin lesions or observed behavior.

## Methods

### Search

A systematic review was conducted in April 2020 using the search engine Web of Science due to its wide range of source databases (41). The Web of Science default time span of 1900–2020 was applied. The search terms “magnesium,” “pig,” “swine,” “livestock,” “behavior,” “aggression,” and “stress” were used in combination using the Boolean operators. The search term string used was “(magnesium OR mg) AND (behavior OR behavior OR stress OR aggression OR aggressive OR cortisol) AND (pig OR pigs OR swine OR porcine OR livestock).”

The references of the final corpus were checked to ensure no literature was missed. Five further studies were found; however, one was a conference abstract (42) and three were not accessible (43–45) and, therefore, are not included in this review. The final paper found in the reference check was included in the final corpus (46).

### Inclusion and Exclusion Criteria

**Figure 1:**
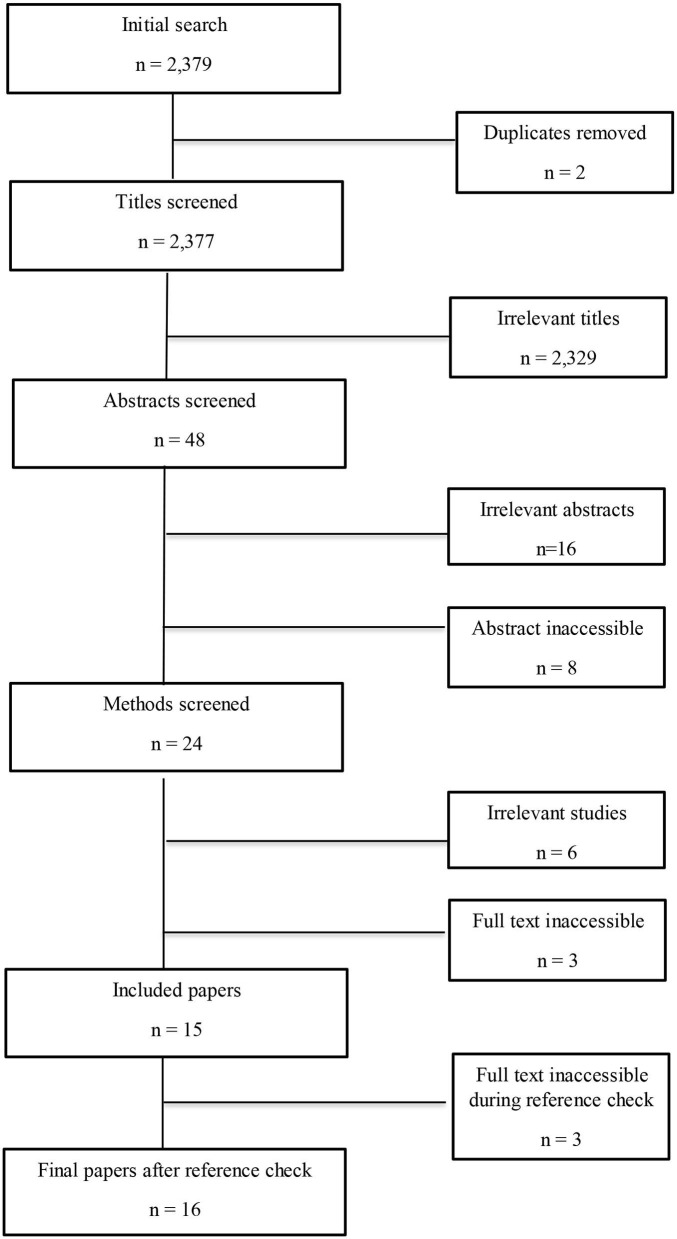
Flowchart to show the study selection process. Irrelevant studies included those that did not have pigs as their study species or include a measures of stress.

Duplicates were removed and the remaining papers were filtered in four stages: (1) title; (2) abstract; (3) methods; and (4) full paper. Papers were included if: (1) pigs were the main study species, with a focus on the whole live animal; and (2) the study included dietary magnesium and at least one measure of stress. Papers were excluded if they were: (1) review papers; (2) conference abstracts; (3) *in vitro*; or (4) research not including a magnesium supplement or a measure of stress. Papers were also excluded if the abstract or full text could not be accessed or was not in English (Figure 1).

### Information Extraction

The following information was extracted from the final remaining papers: (1) aim of study; (2) sample size, sex and age of individuals or stage of production; (3) genotype; (4) experimental treatment(s); (5) dietary treatments (type of magnesium supplement, dose, administration method); (6) measured outcomes of stress; and (7) results.

## Results

### Characteristics of Included Studies

The initial search identified 2,379 studies that were filtered according to the inclusion and exclusion criteria (as defined in section Inclusion and Exclusion Criteria), resulting in a final corpus of sixteen papers (Figure 1 and Table 1).

**Table 1:** Summary of extracted information for the final review corpus.

	**Weaner**	**Grower**	**Finisher**	**Halothane-genotype**	**Physiological measures**	**Behavioral measures**	**Supplement dose < 1 g**	**Supplement dose 1–5 g**	**Supplement dose 5–10 g**	**Supplement dose > 10 g**	**Reduction in stress measure?**
Apple et al. (47)			x	x	x					x	
Caine et al. (48)			x	x		x	x				
D'Souza et al. (46)			x		x					x	
D'Souza et al. (49)			x		x		x				x
Ehrenbergt and Helbig, (50)	*Not reported*				x		x				x
O'Driscoll et al. (39)		x			x	x				x	x
O'Driscoll et al. (40)		x			x	x	x				x
Otten et al. (51)			x	x	x		x				x
Panella-Riera et al. (52)			x	x		x		x			x
Panella-Riera et al. (53)			x	x		x		x			
Peeters et al. (54)			x	x	x	x		x			x
Peeters et al. (55)			x	x	x	x		x			x
Porta et al. (56)			x		x			x			x
Tang et al. (57)			x		x			x			
Tang et al. (58)			x		x			x			
Tarsitano et al. (59)			x		x			x	x		x
Total %	0	12.50	81.25	43.75	81.25	43.75	31.25	50.00	6.25	18.75	62.50

*All supplement doses were converted into grams by the author*.

Five studies included male and female pigs, seven only used male animals, and four did not report the sex of the animals used (50, 54–56). Sample sizes (including all treatments and controls) across the studies were highly variable, ranging from 10 to 448 pigs in total (average sample size of 124 with a standard deviation of 150). Thirteen of the 16 studies focussed on the effect of magnesium in the finishing phase (~50 kg to slaughter) and two in the grower phase (~20–50 kg live weight); one study did not specify the stage of production or age of the pigs used (50).

Six studies used Large White x Landrace pigs, three used a combination of Landrace, Large White, and Pietrain breeds, two used Pietrain x Hypor animals, and one used only Landrace and one a Duroc x Large White x Yorkshire. Two studies did not specify breed, only that the animals were halothane gene positive or negative (47, 48).

Seven studies chose to include pigs that expressed or carried the halothane gene (48, 50–55). This genotype results in the pigs being more susceptible to porcine stress syndrome, a genetic condition characterized by stress induced hypothermia (60). Three of these studies compared groups of pigs positive for the halothane gene with animals either negative (52, 53) or carriers (48), whereas both studies by Peeters et al. (54, 55) used only carriers of the gene and one study did not state the genetic profile of the animals used (50).

### Treatments

#### Dietary Treatment

A total of 10 different magnesium supplements were used across the 16 studies. Four supplements were used in multiple studies; magnesium acetate was used by both Peeters et al. (54) and Peeters et al. (55). Two studies used magnesium-rich marine algae extract with a magnesium level of 59,520mg/Kg (39, 40) and two used magnesium sulfate (46, 53). Magnesium aspartate, also known as magnesium aspartate hydrochloride, was another popular choice with six studies choosing to use this supplement (46, 48–50, 56–58). Other magnesium supplements were magnesium mica (47), magnesium fumarate (51), magnesium carbonate (52), magnesium oxide (59), and magnesium chloride (46). The dose varied greatly between studies with 20 different doses administered. The majority of studies included magnesium at a level of < 1 g (31.25% of the studies) or between 1 and 5 g (50.00% of the studies). Only one used a dose between 5–10 g and three > 10 g (Table 1). Six studies compared two or more different amounts of the specific magnesium supplement (48, 49, 51, 56, 58, 59). There were 10 different durations of supplementation ranging from 2 to 115 days (average of all durations in each study was 22.24 days with a standard deviation of 33.65 days). One study supplemented during a liveweight range (30–100 kg) rather than days (51), and two studies compared long and short-term supplementation (48, 56). Two different supplementation methods were used. Thirteen studies opted to add the supplement to the pigs standard feed, a further two added it to drinking water (54, 55) and one supplemented both feed and water depending on the length of application (56).

#### Methods of Inducing and Measuring Stress

Stress was often induced by slaughter (48, 51–53, 59), and measured in terms of behavior and skin lesions in or following the lairage period (48), handling and stunning procedures (46, 52, 53), or blood parameters following slaughter (51, 56, 59). Transport, an acute stressor, was included in multiple studies (47, 54–58) during which some were transported within their original groups (47); some were mixed and then transported (55) and some experienced a transport simulation (54). Others used common stressors experienced on a commercial farm, such as regrouping (39, 40), withdrawal of feed (39), handling technique (46, 49), or exercise (50).

A total of 13 studies used physiological measures to quantify stress and six used behavioral measures with four studies employing both techniques (Table 1 and Supplementary Table 1). Stress was typically assessed by measuring cortisol, with seven studies using plasma or serum (51, 56–59) and three using salivary cortisol (39, 40, 54). Other physiological measures used to quantify stress included norepinephrine levels in two studies (49, 51), adrenaline and noradrenaline (46) and one study measured tachycardia and hyperventilation (50). The level of aggression or harmful behaviors was assessed using behavioral observations in six studies (39, 40, 48, 52–54). Lesion scores were used in a further four studies (39, 40, 53, 55).

### Outcomes of Included Studies

Of the final corpus of studies (Table 1), 10 found that supplementary magnesium significantly reduced at least one measure of stress. A further two studies found supplementary magnesium reduced serum cortisol levels, although not significantly (57, 58). Two studies found supplementary magnesium resulted in a statistically significant increase in stress (48, 53) suggesting that it may be harmful in some instances. Two studies found no difference in measures of stress between dietary treatments. Apple et al. (47) showed that 25 g/Kg magnesium mica had no effect on stress and similarly, D'Souza et al. (46) found no significant difference between a control diet and three different magnesium-supplemented diets on adrenaline and noradrenaline.

Sample size or power calculations were not reported and the total number of animals used in the 16 studies ranged from 10 to 448 with eight studies using between 1 and 50 pigs, two using 51–100 and five having a total sample size of over 100 animals (Table 1). Six of the 15 studies appear to have less than 10 animals per treatment group (including dietary, genotype, and stressor treatments) (47, 50–52, 57, 58). Thus, the results from studies with a low sample size should be interpreted with caution.

#### Cortisol and Physiological Measures

Salivary cortisol was reduced in two studies (39, 40) and plasma or serum cortisol in three (51, 56, 59). A further two studies found magnesium aspartate reduced serum cortisol concentrations; however, these were non-significant trends (57, 58). Porta et al. (56) found mixed results depending on the length of time and application method. They observed that serum cortisol was decreased in pigs receiving 5 mg/Kg of magnesium aspartate hydrochloride in feed for 115 days; however, if magnesium was administered at a higher level (40 mg/Kg) in water for 5 days before slaughter serum cortisol was increased in comparison to the control. Peeters et al. (54) also added magnesium to water and found pigs receiving magnesium acetate at 3 g/L for 2 days before a transport stressor resulted in salivary cortisol level not returning to baseline as quickly as in control pigs, suggesting that magnesium did not positively influence stress.

O'Driscoll et al. (39) showed that during the regrouping stressor, supplemented females had lower cortisol levels than control females; however, during a 21 h feed withdrawal, there was no significant difference in salivary cortisol between dietary treatments. In a second study (40) magnesium also significantly lowered salivary cortisol levels in standard housing conditions.

Other physiological measures were also used to measure stress. D'Souza et al. (49) showed that overall, boars fed with supplementary magnesium aspartate had significantly lower plasma norepinephrine than pigs that received the control diet. Ehrenbergt and Helbig (50) over a 24 h period after stress caused by running on an ergometer.

#### Behavior

Magnesium was found to have a beneficial influence on aggressive or harmful behaviors in three studies including reduced duration (but not frequency) of aggressive behaviors (40), and pigs being slower to perform the first retreat attempt in the abattoir CO_2_ stunning unit (52). Two found no effect of magnesium in the diet on behavior (39, 53). Caine et al. (48) found supplementing feed with 40 mg/Kg of magnesium aspartate hydrochloride for 7 days resulted in an increase in aggressive behaviors, although a long-term low-level of magnesium in the diet (magnesium aspartate hydrochloride 5 mg/Kg in feed for 43 days before slaughter) had no effect. In another study, when pigs were placed in a vibration crate designed to simulate transport the magnesium-supplemented pigs were visibly calmer and spent more time lying down (54).

#### Skin Lesion Scores

All but one of the studies measuring lesion scores found reduced lesions in supplemented pigs in comparison to the control (39, 40, 55). Panella-Riera et al. (53), on the other hand, found the opposite effect. This study found that pigs had more severe skin lesions (typically due to biting during an aggressive encounter) when they received a diet containing elemental magnesium (1.2 g/Kg) in combination with L-tryptophan (8 g/Kg). Peeters et al. (55) found skin lesions in the loin area were reduced.

#### Halothane Genotype

Although now bred out of commercial pig herds, many studies in this review focus on halothane-genotype pigs. Two studies found that halothane-genotype pigs responded positively to increased dietary magnesium, evidenced by pigs taking longer to show the first retreat attempt in the abattoir stunning unit (53) or reduced hyperventilation and tachycardia following transport stress (50). One study showed no difference between genotypes (52); however, others found that halothane-genotyped pigs had higher plasma norepinephrine (51) and aggressive behaviors were more frequent in pigs carrying the halothane gene in comparison to control or non-halothane-genotype individuals (48). The final two studies involved only pigs that carried the halothane genes and so no comparison could be made between these and individuals with a different genotype (54, 55).

## Discussion

The aim of this systematic review was to examine the current scientific literature exploring the use of magnesium to reduce stress in pigs. Sixteen studies, published between the years 1991 and 2013, met the inclusion criteria. Ten of these reported at least one positive significant effect of supplementary magnesium on physiological measures of stress and/or measures of harmful or aggressive behavior (Table 1). Not all studies found supplementary magnesium to be beneficial. Including Caine et al. (48) who found that short-term, high doses of magnesium (40 mg/kg for 7 days) increased the frequency of aggressive behaviors, and Panella-Riera et al. (53) who reported that the carcases of pigs fed for 5 days before slaughter on a diet supplemented with 1.2 g of elemental magnesium and 8 g of L-tryptophan had an increased number of skin lesions, suggesting they were more active or fought more during the transport or slaughter period. In both studies, supplementary magnesium was only given for a very short period of time, 5 and 7 days before slaughter, respectively.

A common theme throughout this literature was porcine stress syndrome, a genetic condition caused by the halothane gene that is characterized by hypothermia induced by stress (60) which can often result in sudden death and poor meat quality. In the UK, the halothane gene has now been removed from commercial pig production through genetic selection, rendering the results from these studies less relevant to current UK commercial pig production, although they may remain relevant to pig production in other countries. Overall, the results of the seven studies focusing on porcine stress syndrome susceptible pigs, suggests that magnesium supplementation in some cases may have a positive impact on animals that are genetically susceptible to stress (Supplementary Table 1). Alternatively, if focusing on the nine studies that did not include halothane-genotype pigs, five studies found at least one measure of stress was improved when the pigs received magnesium. One of these five studies, however, also showed that magnesium increased serum cortisol levels when given at a low-level for a longer period of time (56). A further three found no significant effect. This suggests that more research to determine appropriate dose regimens is required.

There is also large amount of literature examining how magnesium may improve meat quality, although not all studies include measures of stress (61–63). Thirteen of the 16 studies retrieved in this review were concerned with the effects of magnesium on meat and thus discussed measurements of stress from the perspective of improving pork quality. These studies also tended to focus only on the end stage of the commercial pig's life; for example, both Apple et al. (47) and Porta et al. (56) focused on transport and slaughter stress. Although the later stages of the pigs' lifetime may seem like the most obviously stressful period, stress is likely to occur at various points throughout the whole life and may have a cumulative impact on welfare and performance. Therefore, it would be worthwhile to explore further, the effect of including magnesium during earlier life stages.

Throughout the literature, cortisol was the most common measurement taken to indirectly assess levels of stress. Cortisol was measured either in the plasma, serum, or saliva with concurrent recording of behavioral measures including the frequency and duration of aggressive behavior (Table 1). Cortisol is an easy to obtain measure of arousal or stress and so it is unsurprising that so many of the studies used cortisol measures. However, cortisol is highly variable even within an individual, and can be elevated due to both positive and negative arousal; as such, cortisol measures may be more interpretable when contextualized with behavioral responses that can help to infer the valence of the response (6).

Although measures of cortisol and behavior were common across the studies, in terms of the nutritional treatment, there was a lack of consistency between methodologies with often no clear reasoning for the doses, durations, or types of magnesium used. As shown by the number of studies extracted in this review, this is a relatively new nutritional method that is yet to achieve scientific consensus on when and how it may be most beneficial, or even harmful. Cost will be key in terms of farmers' willingness to implement a new strategy. Investing in additional magnesium will need to be cost effective and worthwhile for the producer, either because the magnesium is a cheap strategy to implement or stress is reduced in a large enough proportion of the livestock (with clear benefits, such as improved performance), to make the treatment a worthwhile investment. Based on the studies in this review, there appears to be no clear conclusion regarding the best method to administer supplementary magnesium in order to reduce stress and further research should strive to validate appropriate dosage, duration, and application of magnesium.

Despite the inconsistency between methodologies rendering valid comparisons between studies difficult, it is clear from the results that supplementary magnesium can have beneficial effects on reducing measures of stress, aggression, and improve meat quality in pigs of varying genotypes. A large amount of research was focused on the end of the commercial pig's life and although this is a key time in terms of pork quality, it would also be beneficial to investigate further how introducing magnesium into the diet earlier on in life may improve welfare, performance, and other key measures. Overall, there is a limited amount of scientific evidence to support the use of magnesium to reduce aggression and stress on commercial pig farms; however, the weight of the evidence for magnesium supplementation in pigs is positive and more thorough investigation of the impact of magnesium on stress in pigs is merited.

## Data Availability Statement

The original contributions presented in the study are included in the article/Supplementary Materials, further inquiries can be directed to the corresponding author/s.

## Author Contributions

EB conducted the search. EB, LD, and LC wrote and edited the manuscript. All authors contributed to the article and approved the submitted version.

## Conflict of Interest

The authors declare that the research was conducted in the absence of any commercial or financial relationships that could be construed as a potential conflict of interest.
