# Supplementary material for: Is Magnesium Supplementation an Effective Nutritional Method to Reduce Stress in Domestic Pigs? A Systematic Review
Source: Front Vet Sci. 2021 Jan 12;7:596205. doi: 10.3389/fvets.2020.596205 (PMC7835408; doi:10.3389/fvets.2020.596205)
Supplement: Supplementary file 1 [file Table_1.DOCX]

**Supplementary Material**

**Table S1:** Extracted information from included studies including aim, animal information, dietary treatments, outcomes measured and results summary. (+ positive result; - negative result; o no effect)

| **Author** | **Aim of study** | **Genotype, Sex & Age/Stage of Production** | **Experimental Treatment (s)** | **Dietary Treatment (s)** | **Measured outcomes** | **Results** | **Size of Effect** |  | **Results summary** |
| --- | --- | --- | --- | --- | --- | --- | --- | --- | --- |
| **Apple, et al., (2005)** | Effect of magnesium supplementation on performance, transportation stress and meat quality effects | (1) Halothane gene carriers  (2) Mixed sex  (3) Finisher stage  (n=36; Magnesium supplemented = 18) | *Control –* pigs remain in pen.  *Stress –* 3 hours of transport. | *Control*  *Magnesium mica* - 2.5% magnesium mica 71 days in feed | *Cortisol* (blood taken every 30 minutes for 3 hours) | Transport stress treatment significantly increased cortisol levels but there was no significant diet – treatment interaction. | Cortisol decreased by 20.85% in magnesium diet pigs exposed to transport stress in comparison to control diet. |  | o |
| **Caine, et al., (2000)** | Effect of magnesium supplementation at a high or low doses for long or short periods on performance, behaviour, meat quality and carcass composition in pigs with different halothane genotypes | (1) 50% positive for halothane gene, 50% carrier  (2) Mixed sex  (3) Finisher stage  (n=142; magnesium supplemented = 48) | *Lairage and slaughter* | *Control*  *Long term low level* - Magnesium aspartate hydrochloride 5mg/Kg in feed for 43 days before slaughter  *Short term high level* - Magnesium aspartate hydrochloride 40mg/Kg for 7 days in feed before slaughter | *Behaviour* (assessed for 2h, 3 days prior to slaughter and during the 1h lairage period in the abattoir) | Increased aggression in short-term high-dose pigs. Aggression was twice as high in carrier genotype pigs.  Long term low level magnesium had no effect. | Aggression increased by 113.04% and 68.97% in short term high dose pigs in comparison to long term low dose and control diets respectively (3 days prior to slaughter). |  | - |
| **D’Souza, et al. (1998)** | Investigate whether dietary magnesium can improve meat quality. | (1) Large white X Landrace  (2) Male  (3) Finisher stage  (n=48; magnesium supplemented = 24) | *Minimal handling* at the abattoir  *Heavy handling* at the abattoir | *Control*  *Magnesium aspartate 100mg* supplemented in feed 5 days prior to slaughter  *Magnesium aspartate 230mg* supplemented in feed 5 days prior to slaughter | *Plasma epinephrine and norepinephrine* - blood collected at time of exsanguination. | Pigs receiving supplementary magnesium had significantly lower plasma norepinephrine than control pigs but there was no difference in epinephrine.  There was no difference between handling or supplement doses. | A magnesium diet decreased plasma norepinephrine by 50.00% when pigs were exposed to minimal handling at the abattoir. |  | + |
| **D’Souza, et al., (1999)** | Compare the effect of three different magnesium supplements on the pork quality of pigs that are stress before slaughter. | (1) Large White X Landrace  (2) Male  (3) 90Kg – Finisher  (n=48) | *Negative handling in abattoir* - All pigs experienced 15 electric shocks 5 minutes before slaughter. | *Control*  *Magnesium aspartate* *40g*  *Magnesium sulphate* *31.6g*  *Magnesium chloride* *38.3g*  Supplements fed for 5 days prior to slaughter. All magnesium diets are equal to 3.2g elemental magnesium per magnesium diet. | *Plasma noradrenaline and adrenaline* - blood collected at time of exsanguination. | No significant difference found in plasma adrenaline and noradrenaline between diets. | Adrenaline SED – 0.896  Noradrenaline SED – 0.522 |  | o |
| **Ehrenbergt, et al, (1991)** | Can dietary magnesium reduce the effects of porcine stress syndrome? | (1) Landrace  (2) Sex not reported  (3) Age not reported  (n=10; magnesium supplemented = 10) | *Stress* - run on an ergometer at 1.3m/s for 10 minutes. | *Magnesium aspartate hydrochloride 40mg* per Kg in feed for 2 days | *Respiratory rate*  *Rectal temperature*  *Heart rate*  *Blood* - sampled before and over 24h after stressors | Magnesium reduced hyperventilation and tachycardia after stress over a 24h period.  Glucose and lactate were significantly reduced during and after stress. | Not reported |  | + |
| **O’Driscoll, et al. (2013a)** | Does magnesium from an organic source (marine algae) improve the welfare of undocked pigs? | (1) Large white X Landrace  (2) Mixed sex  (3) 4 - 20 weeks  (n=448; magnesium supplemented = 224) | *Mixing* - 56 days  *Out of feed event* - at day 112, where all pigs had no access to food for 21 hours. | *Control*  *Marine algae extract (Mg – 59,520ppm)* 5% in feed throughout study (92 days).  (Separate male and female control and supplement groups) | *Salivary cortisol -* collected from focal pigs 1 day before and 2 days after the mixing and out of feed events  *Skin and tail lesions -* recorded for focal pigs 1 day before and 2 days after the mixing and out of feed events  *Behaviour -* recorded after mixing and during out of feed event | *Mixing*: Salivary cortisol was lower in supplemented females than control females. No effect of supplement on aggressive and harmful behaviours or number of skin or tail lesions  *Out of feed event*: No effect of diet on behaviour. No effect of diet on salivary cortisol. Supplemented pigs had less body lesions overall | Female supplemented pigs on average had salivary cortisol levels that were 20.94% lower than control pigs during mixing.  Control animals had 24.24% higher lesion scores than control pigs during the out-of-feed event. |  | + |
| **O’Driscoll, et al. (2013b)** | Does magnesium from an organic (marine algae) source improve welfare of growing pigs? | (1) Large white X Landrace  (2) Mixed sex  (3) 4 – 21 weeks (focus on grower /finisher period)  (n=448; magnesium supplemented = 224) | *Mixing* - on day 56 | *Control*  *Marine algae extract (Mg – 59,520ppm) 0.05%* in feed throughout study (63 days)  (Separate male and female control and supplement groups) | *Salivary cortisol -* collected from focal pigs between days 46-56 and on the same days as the lesion scores were recorded  *Tail and skin lesions –* recorded for focal pigs on multiple days  *Behaviour –* of focal pigs continually observed for 5 minutes each morning and afternoon | No change in frequency of aggressive/harmful behaviours but the duration was significantly less for supplemented pigs.  No effect of diet on tail lesions. Female supplemented pigs had significantly lower skin lesions than other pigs. Supplemented pigs had lower shoulder and ear lesion scores and salivary cortisol. | Duration of aggressive/harmful behaviours was 8.33% less for the supplemented group in comparison to the control.  Salivary cortisol was 11.56% lower in supplemented pigs. |  | + |
| **Otten, et al. (1995)** | Investigate the effects of dietary magnesium on stress, blood metabolites and meat quality in different genotype pigs. | (1) 18 landrace, 18 pietrain. (18 were halothane gene positive)  (2) 24 males & 12 females  (3) Finisher stage  (n= 36; magnesium supplemented = ~24) | *Slaughter* | *Control*  *Magnesium fumarate 10g/kg* in feed from 30kg – 100kg live weight  *Magnesium fumarate 20g/kg* in feed from 30kg – 100kg live weight | *Plasma cortisol, epinephrine and norepinephrine* - blood samples at 35Kg, 57Kg and 87Kg. | Both 10g/kg and 20g/kg of supplementary magnesium reduced plasma cortisol and norepinephrine  Magnesium supplementation had no significant impact on plasma epinephrine concentration | Norepinephine was decreased by 31.94% and 18.85% when dietary magnesium as added at 10g and 20g respectively in comparison to the control.  Cortisol was decreased by 30.67% and 32.91% dietary magnesium as added at 10g and 20g respectively in comparison to the control. |  | + |
| **Panella-Riera, et al., (2008)** | Do natural tranquilisers (magnesium) have different effects depending on the pigs’ genotype. | (1) Landrace, large white and pietrain. 34 halothane gene positive and 27 negative  (2) Male  (3) Finisher stage  (n=61; magnesium supplemented = ~20) | *CO_2_ stunning and slaughter* | *Control*  *Magnesium carbonate 1.28g/kg* in feed 5 days prior to slaughter | *Behaviour -* on the raceway before entering the CO_2_ stunning unit and in the decent to the pit. | In halothane gene negative pigs, magnesium supplemented pigs took longer to attempt the first retreat in the stunning unit. The opposite occurred in halothane gene positive pigs.  All other behavioural results were non-significant | Retreat attempts in the CO_2_ stunning unit were 2.51% lower when pigs were halothane gene negative in comparison to halothane gene positive. |  | + |
| **Panella-Riera, et al., (2009)** | Investigate the effects of supplementing with magnesium with tryptophan on meat quality, feed intake, mortality and behaviour in pigs with different genotypes. | (1) 33 halothane positive (large white and landrace). 33 halothane negative (pietrain)  (2) Males  (3) Finisher stage  (n=69; magnesium supplemented = ~23) | *CO_2_ stunning and slaughter* | *Control*  *Elemental magnesium 1.2g/kg and 8g L-tryptophan 8g/kg* in feed 5 days prior to slaughter | *Behaviour* - recorded in the corridor of the abattoir before stunning and during exposure to CO_2_. | No difference in feed intake or in behaviour in the abattoir.  Magnesium supplemented pigs had more severe skin lesions. | Magnesium and tryptophan diet resulted in a 494.64% increase in “severe skin damage” and a 125.23% increase in “skin damage effecting quality” in comparison to a control diet. “Slight skin damage” and “no skin damage” decreased by 69.96% and 100% respectively. |  | - |
| **Peeters, et al., (2005)** | Effect of magnesium supplementation on stress responses of pigs during transportation. | (1) Peitrain x Hypor (halothane carriers)  (2) Sex not reported  (3) Finisher stage  (n=126; Mg supplemented= 21) | *Transport simulation* -groups of 3, pigs were subjected to vibration for 2 hours in a vibration crate designed to simulate transport. | *Control*  *Magnesium acetate 3g/L* in water for 2 days. | *Behaviour* - level of restlessness were observed by camera above the vibration station.  *Salivary cortisol* – collected the day before and after treatment and after the recovery period | Magnesium treated pigs spent more time lying down during the second half an hour of vibrations. Supplemented pigs were visibly calmer than controls.  Salivary cortisol levels of magnesium pigs did not return to the level recorded before the stressor as quickly in comparison to other dietary treatments. | Salivary cortisol was 30.36% higher in magnesium diet pigs than the negative control after stress. |  | +/- |
| **Peeters, et al., (2006)** | Effect of magnesium supplementation on stress responses, skin lesion and meat quality. | (1) Peitrain x Hypor (halothane carriers)  (2) Sex not reported  (3) Finisher stage  (n=352; Mg supplemented = 22) | *Transport and slaughter* | *Control*  *Magnesium acetate 3g/L* for 2 days in drinking water | *Plasma cortisol* - 10 control and 10 magnesium supplemented pigs selected for blood sampling  *Skin lesions* – recorded after slaughter | No effect of magnesium on cortisol measurements at slaughter. Magnesium supplemented pigs had fewer loin lesions. | A magnesium diet resulted in 43.06% fewer loin lesions than the control diet. |  | + |
| **Porta, et al., (1995)** | Can magnesium supplementation reduce stress and improve meat quality. | (1) Landrace  (2) Sex not reported  (3) Finisher stage  (n=45; magnesium supplemented = 15) | *Transport and slaughter* | *Control*  *Magnesium aspartate hydrochloride 40mg/Kg* in water 5 days before transport & slaughter.  *Magnesium aspartate hydrochloride 5mg/Kg* in feed for 115 days. | *Serum cortisol, epinephrine and norepinephrine* - blood collected at slaughter | Serum cortisol was lower in high-level short-term magnesium supplementation in comparison to control pigs. Cortisol was higher in low-level long-term magnesium supplemented pigs.  Serum epinephrine was significantly lower in both magnesium groups. | Serum cortisol was reduced by 19.51% when magnesium was given at 40mg for 5 days.  Serum cortisol was increased by 53.66% when magnesium was given at 5mg for 115 days. |  | +/- |
| **Tang et al. (2008)** | Effect of magnesium supplementation on blood parameters and meat quality in relation to transport stress. | (1) Duroc x Large White x Yorkshire  (2) Male;  (3) Finisher stage  (n = 36; magnesium supplemented = 12) | *Control*  *Stress*- 2 hours of transportation. | *Control*  *Magnesium aspartate 1000mg/Kg* 5 days before slaughter  *Magnesium aspartate 2000mg/Kg* 5 days before slaughter | *Serum cortisol* - blood was collected during slaughter. | Magnesium decreased serum cortisol levels but not significantly. | In the transport before slaughter treatment group there was a 14.98% and 17.90% decrease in serum cortisol when supplemented with 1000mg/Kg and 2000mg/Kg respectively |  | o |
| **Tang, et al., (2009)** | Effect of magnesium supplementation on blood parameters and meat quality in relation to transport stress. | (1) Large White x Landrace  (2) Male;  (3) Finisher stage  (n = 24; magnesium supplemented = 12) | *Control* – no transport  *Stress* -1.5h of transportation. | *Control*  *Magnesium aspartate 1000mg/Kg* in feed 9 days before slaughter | *Serum cortisol* - blood was collected immediately after stressor or non-stressor | Non-significant trend for magnesium supplemented pigs to have lower serum cortisol | When exposed to the transportation treatment, the magnesium diet showed a 15.48% decrease in serum cortisol in comparison to the control. |  | o |
| **Tarsitano, et al. (2013)** | Evaluate the effects of magnesium supplementation 7 days before slaughter on meat quality and performance. | (1) Landrace x Large White  (2) Male  (3) Finisher stage  (n = 48; magnesium supplemented = 36) | *Transport and slaughter* | *Control*  *Magnesium oxide 0.2%* for 7 days in feed.  *Magnesium oxide 0.4%* for 7 days in feed.  *Magnesium oxide 0.6%* for 7 days in feed. | *Plasma cortisol* - blood was collected immediately after slaughter | Supplementary magnesium decreased the concentration of plasma cortisol concentration. | Serum cortisol was decreased by 23.68% in comparison to the control when magnesium was included at 0.4% or 0.6%.  Serum cortisol was increased by 13.58% when magnesium was included at 0.2% |  | + |
